# Prognostic value of hepatorenal function following transcatheter edge-to-edge mitral valve repair

**DOI:** 10.1007/s00392-021-01908-w

**Published:** 2021-07-12

**Authors:** Tetsu Tanaka, Refik Kavsur, Maximilian Spieker, Christos Iliadis, Clemens Metze, Patrick Horn, Atsushi Sugiura, Stephan Baldus, Malte Kelm, Georg Nickenig, Roman Pfister, Ralf Westenfeld, Marc Ulrich Becher

**Affiliations:** 1 Department of Medicine II, Heart Center Bonn, University Hospital Bonn, Venusberg-Campus 1, 53127 Bonn, Germany; 2 Department of Cardiology, Heart Center, University Hospital Düsseldorf, Düsseldorf, Germany; 3 Department of Cardiology, Heart Center, University Hospital Cologne, Cologne, Germany

**Keywords:** Mitral regurgitation, Transcatheter mitral valve repair, Hepatorenal dysfunction, MELD-XI score

## Abstract

**Background:**

Hepatorenal dysfunction is a strong prognostic predictor in patients with heart failure. However, the prognostic impact of the hepatorenal dysfunction in patients undergoing transcatheter mitral valve repair (TMVR) has not been well studied.

**Methods:**

In consecutive patients who underwent edge-to-edge TMVR at three German centers, the model for end-stage liver disease excluding international normalized ratio (MELD-XI) score was calculated as 5.11 × ln [serum total bilirubin (mg/dl)] + 11.76 × ln [serum creatinine (mg/dl)] + 9.44. Patients were stratified into high (> 11) or low (≤ 11) MELD-XI score of which an incidence of the composite outcome, consisting of all-cause mortality and heart failure hospitalization, within 2 years after TMVR was assessed.

**Results:**

Of the 881 patients, the mean MELD-XI score was 11.0 ± 5.9, and 415 patients (47.1%) had high MELD-XI score. The MELD-XI score was correlated with male, effective regurgitant orifice area, and tricuspid regurgitation severity and inversely related to left ventricular ejection fraction. Patients with high MELD-XI score had a higher incidence of the composite outcome than those with low MELD-XI score (47.7% vs. 29.8%; *p* < 0.0001), and in multivariable analysis, the high MELD-XI score was an independent predictor of the composite outcome [adjusted hazard ratio (HR) 1.34; 95% confidence interval (CI) 1.02–1.77; *p* = 0.04). Additionally, the MELD-XI score as a continuous variable was also an independent predictor (adjusted HR 1.02; 95% CI 1.00–1.05; *p* = 0.048).

**Conclusions:**

The MELD-XI score was associated with clinical outcomes within 2 years after TMVR and can be a useful risk-stratification tool in patients undergoing TMVR.

**Supplementary Information:**

The online version contains supplementary material available at 10.1007/s00392-021-01908-w.

## Background

The cardiovascular system is in close relation to other organ systems, including the kidney and the liver, and impairment of cardiac function can lead to hepatic or renal dysfunction and vice versa [1–3]. The interactions of the heart with the liver and kidney are named cardiorenal and cardiohepatic syndromes. It has been reported that the presence of hepatic or renal dysfunction is a strong predictor of adverse clinical events in patients with heart failure (HF). Therefore, scoring models of renal and liver dysfunction have been required for assessing risk in patients with HF. The Model for End-stage Liver Disease excluding international normalized ratio score (MELD-XI) is one of the scoring models that have been widely used for assessment of renal and hepatic function [4]. The MELD-XI score reflects liver and renal function and is calculated based on serum total bilirubin and creatinine. In addition, previous reports showed the prognostically predictive value of the MELD-XI score in patients with HF [5–8].

Transcatheter edge-to-edge mitral valve repair (TMVR) is an emerging treatment option for symptomatic severe mitral regurgitation (MR) [9, 10]; however, according to the latest dataset from the Transcatheter Valve Therapy Registry [11], an incidence of adverse clinical events is still high even after TMVR. Moreover, patients with MR usually have multiple risk factors and comorbidities that can affect their clinical outcomes. Therefore, a simple and useful tool for risk stratification is required for patient selection in TMVR. Previous studies showed that hepatic or renal dysfunction was associated with worse clinical outcomes in patients with TMVR [12, 13]; however, the prognostic value of the combined marker of hepatorenal dysfunction has not been fully evaluated. In the present study, we investigated the association between MELD-XI score and clinical outcomes after TMVR.

## Methods

### Study population

This study was designated as a retrospective analysis from the Heart Failure Network Rhineland registry, which is a multicenter, prospective, observational registry of symptomatic patients with MR who undergo TMVR at three high-volume centers in Germany (University Hospitals of Bonn, Cologne, Duesseldorf) [14, 15]. From this registry, we identified consecutive patients who underwent their first edge-to-edge TMVR with the MitraClip system (Abbott Vascular, Santa Clara, California) between October 2011 and October 2018. Patients with available pre-procedural serum creatinine and total bilirubin results were included in the analysis. We excluded patients who were lost to follow-up within one month after TMVR. Patients agreed to participate in our registry which was approved by the Ethical Committee of the individual center in accordance with the Declaration of Helsinki.

### Procedure

The indication for TMVR was moderate-to-severe or severe MR accompanied by symptomatic HF according to the New York Heart Association (NYHA) functional classification in patients considered as inoperable or at high surgical risk. After a standardized diagnostic workup including transesophageal echocardiography (TEE), the decision to perform the intervention was taken by the interdisciplinary heart team of each center. Procedures were performed under general anesthesia or sedation with three-dimensional TEE and fluoroscopic guidance. Details of the device system and procedure have previously been well described [16]. The discretion of whether a second or third device needed to be used was left up to the treating physicians. Device success was defined as post-procedural MR ≤ 2 and post-procedural mean transmitral valve pressure gradient < 5 mmHg after deployment of one or more clips to achieve leaflet approximation and retrieval of the delivery system.

### Assessment of hepatorenal function

Hepatorenal function was assessed using the MELD-XI score which was calculated as 5.11 × ln (serum total bilirubin in mg/dl) + 11.76 × ln (serum creatinine mg/dl) + 9.44 [4]. The creatinine and total bilirubin measurements were performed at each institutional laboratory with other parameters, including N-terminal pro-B-type natriuretic peptide (NT-proBNP) and hemoglobin. A high MELD-XI score was defined as > 11, according to a previous report [5, 7]. Post-procedural blood data, including creatinine and total bilirubin, were collected at 1- and 12-month follow-up after TMVR. In addition, anemia was defined according to the World Health Organization criteria (hemoglobin < 13 g/dl for male patients; hemoglobin < 12 g/dl for female patients).

### Echocardiographic parameters

We assessed echocardiographic parameters performed at baseline and discharge according to the current guidelines [17]. The severity of MR was graded as follows: grade 0, none; 1 + , mild; 2 + , moderate; 3 + , severe. All measurements were reviewed by an independent cardiologist dedicated to echocardiographic evaluation at each center.

### Clinical follow-up

The primary endpoint was a composite outcome, consisting of all-cause mortality and hospitalization due to worsening HF within 2 years after TMVR. As secondary endpoints, all-cause mortality and hospitalization due to worsening HF within 2 years after TMVR were examined separately. All suspected adverse events were independently adjudicated by the local heart team according to the criteria of the Mitral Valve Academic Research Consortium 2 [18]. The need for hospitalization due to worsening HF was determined based on the attending physicians’ discretion without any prespecified criteria. The occurrence of clinical events was recorded from admission and outpatient medical records. Telephone interviews were also performed with the patients’ general practitioners or family. In addition, HF medication, including beta blockers, renin–angiotensin system (RAS) inhibitors, and aldosterone antagonists, and dosage with a standardized furosemide equivalent were recorded at baseline [19].

### Statistical analysis

Continuous variables were tested for normal distribution using the Kolmogorov–Smirnov test. Normally distributed variables are presented as the mean ± standard deviation and compared using *t* tests. In contrast, non-normally distributed variables were expressed as medians with an interquartile range (IQR) and compared between groups using the Mann–Whitney *U* test. Categorical data were presented as numbers and percentages, and the differences between groups were evaluated using the chi-square test. Paired *t* test or Wilcoxon signed rank test compared change in hepatorenal function after TMVR within the same patient. Logistic regression analysis was performed to detect parameters that were related to the MELD-XI score. The variables with *p* < 0.05 on univariate analysis were incorporated into a multivariable regression model. The receiver-operating characteristic (ROC) curve was used to assess the validity of the cut-off value of the MELD-XI score for the 2-year composite outcome in the present study. In addition, Harrell’s C-statistic was used to compare the predictive ability of hepatorenal functional markers for the composite outcome by area under the curve analysis, accounting for censoring. Kaplan–Meier cumulative event curves for the composite outcome, HF hospitalization, and all-cause mortality were generated using two groups according to the high or low MELD-XI score. Differences between the groups were compared using the log-rank test. Univariate and multivariable Cox-proportional hazard models were used to calculate the hazard ratios (HRs) with 95% confidence intervals (CIs) for MELD-XI score for the composite outcome within 2 years after TMVR. In univariate analysis, we analyzed the HRs of conventional covariables that were determined according to previous reports. In multivariable analyses, covariates were included that showed significance (*p* < 0.05) in the univariate analyses, considering multicollinearity. A stratified analysis was performed to assess effects of interactions between high MELD-XI score (> 11) and clinical parameters on the composite outcome. The stratified analysis consisted of the following parameters: sex (male vs female), age (≥ 75 years vs < 75 years), etiology of MR (functional MR vs degenerative MR), MR severity at baseline (moderate-to-severe vs. severe), left ventricular ejection fraction (LVEF) (> 30% vs ≤ 30%), tricuspid annular plane systolic excursion (TAPSE) (≥ 15 mm vs < 15 mm), tricuspid regurgitation (TR) severity at baseline (≥ severe vs < severe), device success (yes vs no), and residual MR (≥ Grade II vs < Grade II), and additionally the *p* value for interaction between subgroups and high MELD-XI score (> 11) was examined. In addition, we performed a restricted cubic spline with 4 knots at the MELD-XI score to model a relationship between the MELD-XI score and the composite outcome. Statistical significance was set at *p* < 0.05. All analyses were conducted using Stata 15.1 (StataCorp, College Station, TX, USA) or JMP version 14.0 for Mac (SAS Institute Inc., Cary, NC, USA).

## Results

### Clinical characteristics of the study population

Of 881 patients, the mean age was 77.0 ± 9.0 years and 58.0% were of male sex (Table 1). Eighty-three percent of the patients were classified in NYHA functional class III or IV. The expected surgical mortality rate was elevated, as evidenced by the median logistic EuroSCORE of 16.4% (IQR 9.2–28.9%). Pre-procedural MR severity was moderate-to-severe or severe in 13% and 87% of the patients, respectively. The mean left ventricular ejection fraction (LVEF) was 44.6 ± 15.1%, and functional MR was the etiology of MR in 61% of the patients.

**Table 1:** Baseline characteristics

	All	MELD-XI > 11	MELD-XI ≤ 11	*p* value
	*n* = 881	*n* = 415	*n* = 466	
Age (years)	77.0 ± 9.0	76.8 ± 8.8	77.1 ± 9.1	0.66
Male, *n* (%)	511 (58.0)	304 (72.2)	207 (44.4)	< 0.0001
BMI (kg/m^2^)	26.0 ± 4.7	26.1 ± 4.3	25.9 ± 5.0	0.56
Diabetes, *n* (%)	250 (28.4)	128 (30.8)	122 (26.2)	0.13
Hypertension, *n* (%)	719 (81.6)	336 (81.0)	383 (82.2)	0.66
CAD, *n* (%)	547 (62.2)	288 (69.4)	259 (55.7)	< 0.0001
Prior CABG, *n* (%)	239 (27.1)	129 (31.1)	110 (23.6)	0.02
Prior valve intervention, *n* (%)	110 (12.5)	49 (11.8)	61 (13.1)	0.61
Previous MI, *n* (%)	279 (31.7)	154 (37.1)	125 (26.9)	0.001
Previous stroke, *n* (%)	115 (13.1)	61 (14.7)	54 (11.6)	0.19
Atrial fibrillation, *n* (%)	566 (64.4)	281 (67.9)	285 (61.3)	0.048
NYHA class III/IV, *n* (%)	728 (83.0)	357 (86.4)	371 (80.0)	0.01
Pacemaker, *n* (%)	92 (10.4)	52 (12.5)	40 (8.6)	0.06
ICD, *n* (%)	135 (15.3)	84 (20.2)	51 (10.9)	0.0002
CRT, *n* (%)	85 (9.6)	57 (13.7)	28 (6.0)	0.0001
COPD, *n* (%)	170 (19.3)	84 (20.3)	86 (18.5)	0.50
Logistic EuroSCORE (%)	16.4 (9.2, 28.9)	17.8 (9.5, 31.6)	15.0 (8.9, 27.6)	0.04
Anemia, *n* (%)	488 (55.4)	231 (55.7)	257 (55.2)	0.89
Hemodialysis, *n* (%)	15 (2.7)	15 (5.6)	0 (0.0)	< 0.0001
Laboratory data				
MELD-XI score	11.0 ± 5.9	15.9 ± 4.1	6.6 ± 3.1	< 0.0001
Creatinine (mg/dl)	1.52 ± 0.93	2.03 ± 1.13	1.08 ± 0.28	< 0.0001
Total Bilirubin (mg/dl)	0.63 (0.44, 0.97)	0.87 (0.60, 1.29)	0.52 (0.40, 0.70)	< 0.0001
eGFR (ml/min/1.73m^2^)	48.0 (34.4, 63.1)	36.0 (25.0, 46.9)	60.0 (47.1, 70.0)	< 0.0001
NT-proBNP (pg/ml)	2727 (1371, 5691)	4345 (2217, 8541)	1906 (977, 3428)	< 0.0001
Echocardiographic findings				
LVEF (%)	44.6 ± 15.1	41.4 ± 15.0	47.3 ± 14.7	< 0.0001
LVEF ≤ 30%, *n* (%)	183 (20.8)	115 (27.9)	68 (14.6)	< 0.0001
LVEDV (ml)	131 (98, 180)	149 (109, 192)	118 (91, 160)	< 0.0001
LVESV (ml)	69 (40, 116)	89 (48, 129)	56 (37, 98)	< 0.0001
LA volume (ml)	97 (75, 124)	105 (84, 138)	90 (70, 120)	< 0.0001
Functional MR, *n* (%)	537 (61.0)	262 (63.1)	275 (59.0)	0.21
MR severity				0.48
3 + , *n* (%)	110 (12.5)	55 (11.8)	68 (13.5)	
4 + , *n* (%)	771 (87.5)	360 (88.2)	398 (86.5)	
EROA (mm^2^)	0.28 (0.20, 0.36)	0.30 (0.20, 0.38)	0.25 (0.20, 0.33)	0.02
TR severity				< 0.0001
None/ Mild	397 (45.1)	161 (38.8)	236 (50.6)	
Moderate	279 (31.7)	128 (30.8)	151 (32.4)	
Severe or more	205 (23.2)	126 (30.4)	79 (17.0)	
TAPSE (mm)	18.3 ± 5.1	17.3 ± 4.8	19.3 ± 5.1	< 0.0001
SPAP (mmHg)	50.0 ± 16.9	50.1 ± 17.5	50.0 ± 16.4	0.93
Medication				
Beta blocker, *n* (%)	690 (78.3)	325 (78.3)	365 (78.3)	1.00
RAS inhibitor, *n* (%)	679 (77.1)	303 (73.0)	375 (80.7)	0.008
Aldosterone antagonist, *n* (%)	340 (38.6)	169 (40.7)	171 (36.7)	0.24
Loop diuretic, *n* (%)	654 (74.2)	316 (76.1)	338 (72.5)	0.25
Standardized furosemide equivalent (mg/day)	20 (0, 40)	30 (5, 60)	20 (0, 40)	0.0003

Values shown are either *n* (%), mean ± SD, or median (interquartile range)

*MELD-XI score* model for end-stage liver disease excluding international normalized score, *BMI* body mass index, *CAD* coronary artery disease, *CABG* coronary artery bypass grafting, *MI* myocardial infarction, *NYHA* New York heart association, *ICD* implantable cardioverter defibrillator, *CRT* cardiac resynchronization therapy, *COPD* chronic obstructive pulmonary disease, *eGFR* estimated glomerular filtration rate, *NT-proBNP* N-terminal pro-B-type natriuretic peptide, *EuroSCORE* European system for cardiac operative risk evaluation, *MR* mitral regurgitation, *EROA* effective regurgitant orifice area, *LVEF* left ventricular ejection fraction, *LVEDV* left ventricular end-diastolic volume, *LVESV* left ventricular end-systolic volume, *LA* left atrium, *TR* tricuspid regurgitation, *TAPSE* tricuspid annular plane systolic excursion, *SPAP* systolic pulmonary artery pressure, RAS renin–angiotensin system

Clip implantation failed in 12 patients (1.4%), and device success was achieved in 93.0% of the patients. The mean number of implanted clips was 1.5 ± 0.6 (Supplementary Table 1). Echocardiography at discharge showed that residual MR of less than 2 + was prevalent in 56.0% of the patients.

### Analysis of MELD-XI score

**Fig. 1:**
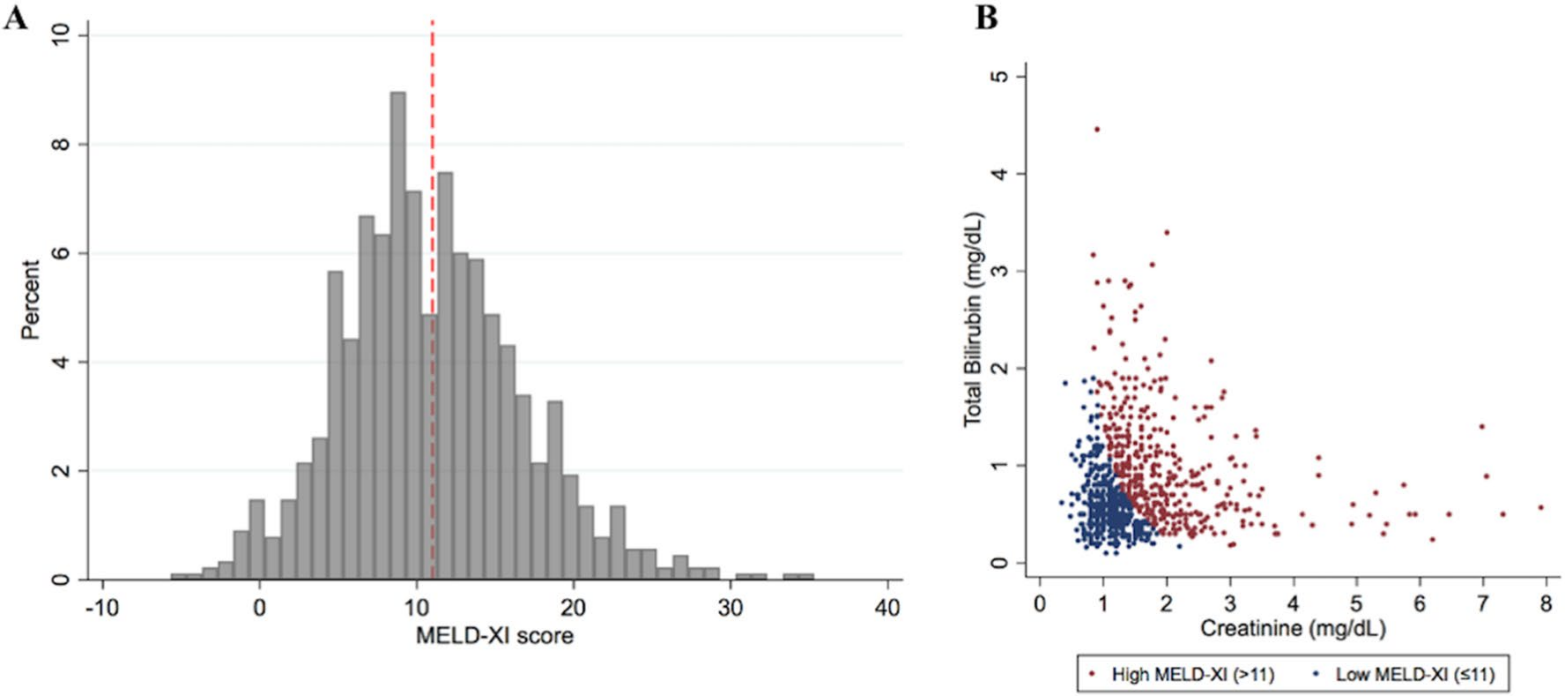
Distribution of the MELD-XI score and the components. The distribution of the MELD-XI score (**A**) and the creatinine and total bilirubin levels that are components of the MELD-XI score (**B**). Dashed red line indicates the MELD-XI score of 11

The mean MELD-XI score was 11.0 ± 5.9 (Table 1), and the distribution of the MELD-XI score is presented in Fig. 1. In addition, the median estimated glomerular filtration rate (eGFR) was 48.0 ml/min/1.73m^2^ (IQR 34.4–63.1 ml/min/1.73m^2^), and the median total bilirubin was 0.63 mg/dl (IQR 0.44–0.97 mg/dl). Fifteen patients received hemodialysis therapies. MELD-XI score was correlated to sex, MR effective regurgitation orifice area (EROA), left ventricular ejection fraction (LVEF), LV end-diastolic volume, TAPSE, right atrial area, left atrial volume, severe or more TR (Table 2). However, on multivariable analysis, male sex had the strongest correlation (standardized *β*: 0.27; 95% CI 0.16–0.37; *p* = 0.003), followed by LVEF (standardized *β*: − 0.19; 95% CI − 0.32 to − 0.26; *p* = 0.004), MR EROA (standardized *β*: 0.15; 95% CI 0.05–0.24; *p* = 0.003), and severe or more TR (standardized *β*: 0.13; 95% CI 0.02–0.22; *p* = 0.02).

**Table 2:** Risk factors for hepatorenal dysfunction (MELD-XI score)

	Univariate analysis			Multivariable analysis		
	*β* coefficient	95% CI	*p* value	*β* coefficient	95% CI	*p* value
Age (years)	− 0.04	− 0.11 to 0.02	0.21			
Male	0.35	0.29–0.41	< 0.0001	0.27	0.16–0.37	0.003
Diabetes mellitus	− 0.05	− 0.12 to 0.02	0.14			
Hypertension	− 0.004	− 0.07 to 0.06	0.91			
EROA (mm^2^)	0.16	0.07–0.25	0.001	0.15	0.05–0.24	0.003
LVEF (%)	− 0.20	− 0.27 to − 0.12	< 0.0001	− 0.19	− 0.32 to − 0.06	0.004
LVEDV (ml)	0.16	0.08–0.24	< 0.0001	− 0.03	− 0.18 to 0.11	0.56
SPAP (mmHg)	0.03	− 0.04 to 0.11	0.36			
TAPSE (mm)	− 0.22	− 0.29 to − 0.15	< 0.0001	0.01	− 0.09 to 0.11	0.91
RA area (mm^2^)	0.17	0.09–0.26	< 0.0001	0.08	− 0.01 to 0.17	0.08
LA volume (ml)	0.16	0.07–0.24	0.0002	0.09	− 0.03 to 0.20	0.15
TR severity: severe or more	0.18	0.11–0.25	< 0.0001	0.13	0.02–0.22	0.02

*MELD-XI score* model for end-stage liver disease excluding international normalized score, *EROA* effective regurgitant orifice area, *LVEF* left ventricular ejection fraction, *LVEDV* left ventricular end-diastolic volume, *SPAP* systolic pulmonary artery pressure, *TAPSE* tricuspid annular plane systolic excursion, *RA* right atrium, *LA* left atrium, *TR* tricuspid regurgitation

Of the 881 patients, 415 patients (47.1%) had high MELD-XI score (> 11), and patients characteristics and echocardiographic findings of each group are shown in Table 1. Patients with the high MELD-XI score (> 11) had a lower LVEF and larger LV end-diastolic volume compared to those with low MELD-XI score (≤ 11) (41.4 ± 15.0% vs. 47.3 ± 14.7%; *p* < 0.0001, and 149 ml (IQR 109–192 ml) vs. 118 ml (IQR 91–160 ml); *p* < 0.0001, respectively). In contrast, there were no significant differences in the rate of device success and length of hospitalization.

Post-procedural changes in the MELD-XI score and other parameters of hepatorenal function were evaluated after TMVR (Supplementary Table 2). Compared to the value at baseline, the median MELD-XI score did not change significantly at 1- and 12-month follow-up after TMVR (11.8 ± 6.3; *p* = 0.86 and 11.2 ± 5.8; *p* = 0.79, respectively).

### Association between MELD-XI score and clinical outcome after TMVR

**Fig. 2:**
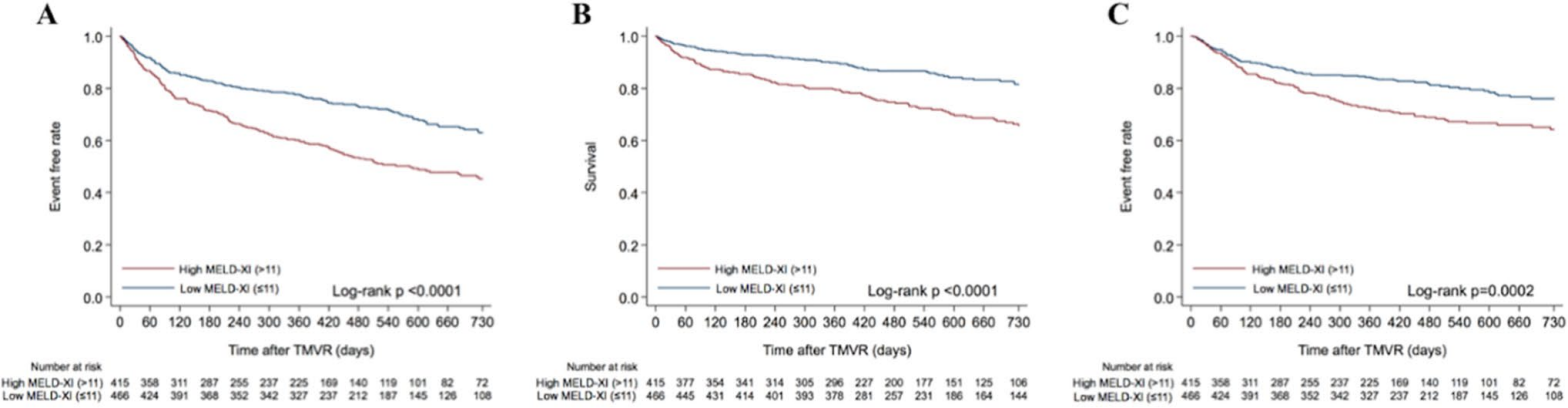
Clinical outcome according to the MELD-XI score within 2 years after TMVR. Kaplan–Meier curves demonstrating clinical outcomes within 2 years after TMVR, including the composite outcome (**A**), all-cause mortality (**B**), and HF hospitalization (**C**), according to the MELD-XI score

Within 2 years after TMVR, 184 patients (20.9%) died, 203 patients (23.0%) were rehospitalized due to worsening HF, and 337 patients (38.2%) experienced the composite outcome (Supplementary Table 1). Patients with the high MELD-XI score (> 11) had a higher incidence of the 2-year composite outcome compared to those with the low MELD-XI score (≤ 11) (54.7% vs. 36.9%; log-rank *p* < 0.0001). The Kaplan–Meier curves for each outcome are shown in Fig. 2. In addition, the high MELD-XI score (> 11) was associated with a higher incidence of the 2-year composite outcome in spite of MR etiology, such as functional MR and degenerative MR (Supplementary Fig. 1).

From the ROC curve for the 2-year composite outcome, the optimal cut-off value of the MELD-XI score was also 11 [area under the curve (AUC): 0.62; *p* < 0.0001] (Supplementary Fig. 2). The Harrel’s C-statistic of MELD-XI score for the 2-year composite outcome was 0.60 (95% CI 0.57–0.63), which was higher than those of eGFR or total bilirubin (C-statistic: 0.57, 95% CI 0.54–0.61; *p* = 0.049, and C-statistic: 0.55; 95% CI 0.52–0.59; *p* = 0.004, respectively) (Supplementary Table 3).

In the multivariable Cox-proportional hazard analysis, covariates were included which showed significance (*p* < 0.05) in univariate analysis (Supplementary Table 4). Patients with high MELD-XI score (> 11) had a higher incidence of the composite outcome within 2 years after TMVR compared to those with low MELD-XI score (≤ 11) (adjusted HR 1.34; 95% CI 1.02–1.77; *p* = 0.04) (Table 3). Moreover, MELD-XI was an independent predictor of the incidence of 2-year composite outcome (adjusted HR 1.02; 95% CI 1.00–1.05; *p* = 0.048).

**Table 3:** Multivariable analysis for association of the MELD-XI score with an incidence of the composite outcome within 2 years after TMVR

	Adjusted HR	95% CI	*p* value
MELD-XI score > 11*	1.34	1.02–1.77	0.04
MELD-XI score per 1 increase*	1.02	1.00–1.05	0.048
Age (years)	1.00	0.98–1.01	0.72
Male	1.20	0.88–1.67	0.24
Diabetes mellitus	1.09	0.83–1.43	0.52
COPD	1.45	1.07–1.95	0.01
CAD	1.04	0.78–1.39	0.81
NYHA class III/IV	1.85	1.25–2.86	0.003
Anemia	1.44	1.08–1.93	0.01
Logistic EuroSCORE (%)	1.01	1.00–1.02	0.048
LVEF ≤ 30%	1.46	1.06–2.00	0.02
LVEDV per 10 ml increase	1.00	0.98–1.03	0.77
TR severity: severe or more	1.37	1.01–1.84	0.04
Standardized furosemide equivalent per 10 mg/day increase	1.02	1.01–1.04	0.02

*Included separately in the multivariable analysis

*MELD-XI score* model for end-stage liver disease excluding international normalized score, *COPD* chronic obstructive pulmonary disease, *CAD* coronary artery disease, *NYHA* New York Heart Association, *EuroSCORE* European System for Cardiac Operative Risk Evaluation, *LVEF* left ventricular ejection fraction, *LVEDV* left ventricular end-diastolic volume, *TR* tricuspid regurgitation

**Fig. 3:**
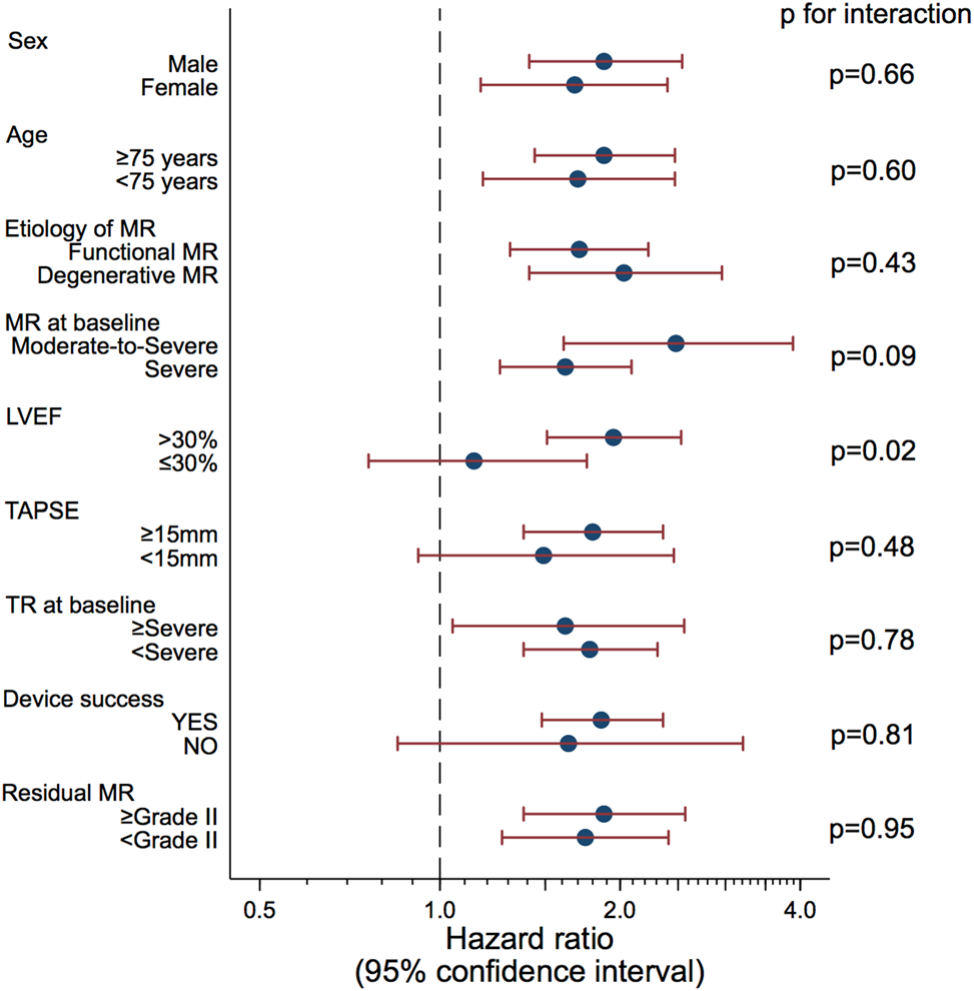
Subgroup analysis of the composite outcome in patients with high MELD-XI score (> 11). A Forest plot illustrates hazard ratios for 2-year composite outcome after TMVR in patients with MELD-XI score (> 11). In each subgroup, hazard ratio and 95% confidence intervals (CIs) are presented. MR = mitral regurgitation; LVEF = left ventricular ejection fraction; *TAPSE* tricuspid annular plane systolic excursion; *TR* tricuspid regurgitation

The association between high MELD-XI score (> 11) and the composite outcome in subgroups is shown in Fig. 3. In the stratified analysis for the composite outcome, there were no significant interactions across the subgroups except for LVEF ≤ 30% (*p* for interaction = 0.02).

**Fig. 4:**
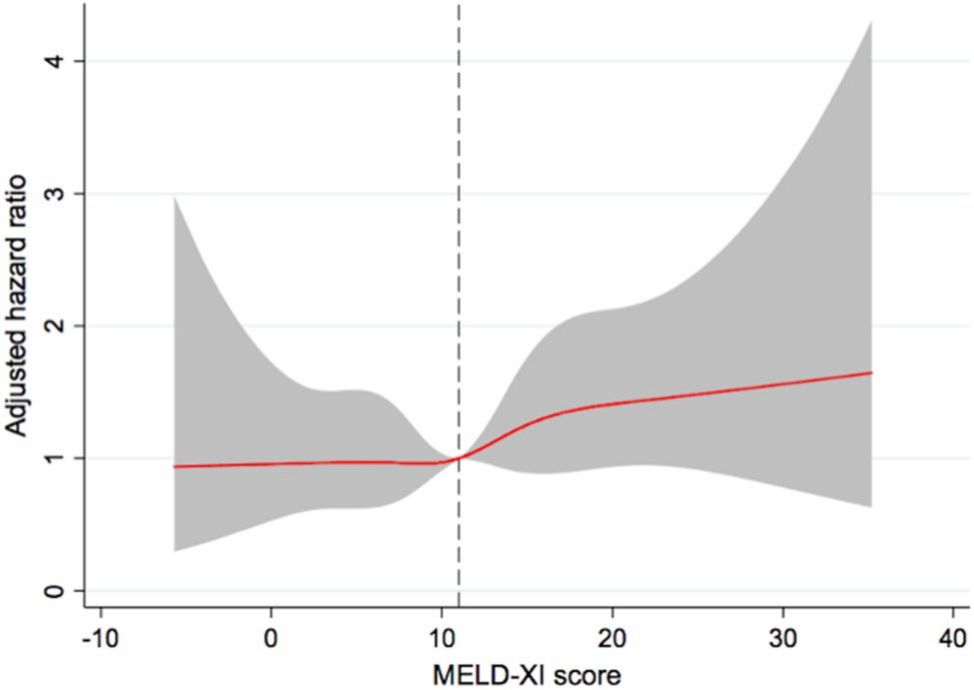
Association between the MELD-XI score and the composite outcome within 2 years after TMVR according to the MELD-XI score. The red solid line indicates the adjusted HR, and the shaded area indicates the 95% CI. The dashed line indicates the MELD-XI score of 11 as reference

Our restricted cubic spline demonstrated a consistent increasing hazard of the composite outcome in the MELD-XI score of greater than 11. However, in the MELD-XI score of less than 11, a significant interaction between the MELD-XI score and the incidence of composite outcome was not observed (Fig. 4).

## Discussion

In the current multicenter study of patients undergoing TMVR, the main findings can be summarized as follows: (1) the MELD-XI score, as both continuous and dichotomous variable, was associated with a higher incidence of the composite outcome, consisting of all-cause mortality and HF hospitalization, within 2 years after TMVR, independent of MR etiology; (2) the MELD-XI score had a higher predictive value of the composite outcome compared to total bilirubin and eGFR; (3) the MELD-XI score was correlated to male sex, MR EROA, and TR severity, and inversely to LVEF; and (4) the risk of the composite outcome increased in the MELD-XI score of greater than 11, whereas the association of the MELD-XI score with clinical outcome was not observed in the MELD-XI score of less than 11.

While both renal and hepatic dysfunction are strong predictors of adverse clinical outcomes in patients with HF [1, 3], the combination of both could increase their ability of risk stratification. The MELD-XI score is one of the most established scoring systems for hepatorenal dysfunction and is an alternative MELD scoring system that excludes international normalized ratio from the calculation. Initially, the MELD scores were developed for patients with hepatic cirrhosis awaiting liver transplantation [4]; however, the predictive value has recently been proven in patients with HF [5, 7, 8]. Furthermore, the MELD-XI score is reported as a risk-stratification tool in patients with valvular interventions [20, 21]. In patients undergoing TMVR, Spieker et al. previously reported the association between the MELD-XI score and all-cause mortality during one-year follow-up in a single-center population [22]. In the present study, we validated the use of this score on a multicenter large-scale cohort of TMVR patients, and additionally assessed the impact of MELD-XI score on the incidence of HF hospitalization. The findings could underline the prognostic impact of hepatorenal function and the utility of the MELD-XI score as a simple tool of risk stratification in patients undergoing TMVR.

The hepatorenal dysfunction related to HF is attributable to elevated cardiac filling pressures or reduced cardiac output, which are named “forward failure” and “backward failure” [23]. In the kidney, the low renal perfusion accompanied by low cardiac output and the renal vein hypertension induced by elevated right-sided cardiac filling pressure lead to neurohormonal activation and increased sodium and water retention, which can be contributable to chronic renal dysfunction. Similarly, in the liver, the decreased hepatic blood flow and the increased hepatic vein pressure can lead to chronic hepatic dysfunction [2, 3]. Accordingly, the association of the MELD scores with cardiac parameters, including the left ventricular systolic function or right atrial pressure, and tricuspid regurgitation has been previously reported [7, 8]. The present study showed that the MELD-XI score in TMVR patients was correlated with male sex, EROA, and TR severity, and inversely with LVEF, which was in line with the previous reports. MR severity is associated with hepatorenal dysfunction, whereas it remains unclear whether the hepatorenal dysfunction can improve after MR correction. Considering that EROA was correlated to the MELD-XI score, the reduction in MR by TMVR might link to an improved hepatorenal function. However, the MELD-XI score did not change significantly after TMVR in the present study, which might be due to the limited number of patients with available follow-up score variables. Further investigation to assess the post-procedural changes in MELD scores are needed in patients undergoing TMVR.

We evaluated the prognostic impact of the MELD-XI score as both dichotomous and continuous variable in TMVR patients. In addition, the predictive value of the MELD-XI score for the composite outcome within 2 years after TMVR was greater than those of eGFR and total bilirubin, separately. The optimal cut-off value of the MELD-XI score is different in various populations of HF [5, 7, 8, 21, 22]. We used one of the cut-off values previously reported and found a similar validity of the cut-off value in the present study. The validity of the cut-off value of MELD-XI score remained consistent among most of subgroups, including those defined according to etiology of MR, MR or TR severity, right ventricular function, device success, and residual MR. In contrast, high MELD-XI score (> 11) was not associated with the composite outcome in patients with LVEF ≤ 30%. Patients with LVEF ≤ 30% had higher MELD-XI score compared to those with LVEF > 30% (12.7 ± 6.0 vs 10.5 ± 5.8; *p* < 0.0001), and, according to ROC analysis, higher cut-off value was optimal for patients with LVEF ≤ 30% (cut-off: 17; AUC: 0.57; *p* = 0.02). Notably, our restricted cubic spline uncovered a continuously increasing hazard ratio of the composite outcome in the MELD-XI of greater than 11, while there was not a significant interaction between the MELD-XI score and the incidence of the composite outcome in the MELD-XI score of less than 11. The results suggest the importance of assessing the MELD-XI score as both dichotomous and continuous variable in clinical settings.

Predictors of long-term clinical outcomes after TMVR has been shown in previous reports from different multicenter registries [24, 25]. In the present studies, LVEF ≤ 30%, chronic pulmonary obstructive disease (COPD), anemia, NYHA class, logistic EuroSCORE, TR severity, and dosage of loop diuretics were associated with the composite outcome within 2 years after TMVR, which was in line with previous reports. COPD is one of the major comorbidities in patients with HF and is associated with increased long-term morbidity and mortality after TMVR [24, 25]. COPD can be a potential trigger for cardiovascular events, including worsening HF, when its reactivation occurs. Moreover, the overlapping symptom of dyspnea with both COPD and HF may lead to misapplication of therapy.

Appropriate patient selection is essential to ensure beneficial effects of TMVR. Controversial results of two randomized control trials, which are COAPT and MITRA-FR trials, highlight the importance of patient selection for TMVR [26]. However, because of the heterogeneity of patients with MR, the patient selection needs demanding assessments of clinical factors, including MR etiology, cardiac function and geometry, and non-cardiac comorbidities. In the present study, we focused on the hepatorenal function and revealed the association between the MELD-XI score and clinical outcomes after TMVR, independently of MR etiology. Furthermore, the MELD-XI score had a higher predictive value for the composite outcome compared to separate evaluations of renal and liver function. Our findings highlight the importance of hepatorenal function in patient selection and risk stratification for TMVR. The MELD-XI score may represent a simple tool for optimization of patient selection for TMVR. However, this study was an observational study without a control group consisting of patients who did not undergo TMVR, and it remains unclear whether TMVR can improve prognosis even in patients with high MELD-XI score. Nevertheless, the MELD-XI score is a scoring system that is calculable from conventional laboratory data and has been widely used in patients with HF [5, 7, 8], which can indicate the adaptability of the MELD-XI score to clinical practice in the field of TMVR.

### Limitations

To the best of our knowledge, this is the first multicentric study assessing the prognostic impact of the MELD-XI score in patients undergoing TMVR; however, several limitations must be acknowledged. First, this was a retrospective study. Therefore, a certain patient selection bias might have impacted our results. Second, we could not distinguish acute hepatorenal injury from chronic dysfunction because we evaluated the MELD-XI score from only the latest laboratory data before TMVR. Finally, we could not assess post-procedural changes in the MELD-XI score after TMVR and could not investigate the prognostic impact of post-procedural changes in the MELD-XI score.

## Conclusion

The MELD-XI score was associated with clinical outcome after edge-to-edge TMVR. Patients with a high MELD-XI score (> 11) had a higher incidence of the composite outcome, consisting of all-cause mortality and HF hospitalization, within 2 years after TMVR compared to those with a low MELD-XI score. The MELD-XI score can be a reliable tool for patient selection and risk stratification in patients undergoing TMVR.

## Supplementary Information

Below is the link to the electronic supplementary material.

Supplementary file1 (DOCX 247 KB)

## Abbreviations

CI: Confidence interval
HF: Heart failure
HR: Hazard ratio
LVEF: Left ventricular ejection fraction
TMVR: Transcatheter mitral valve repair
MELD-XI score: Model for end-stage liver disease excluding international normalized ratio score
MR: Mitral regurgitation
MV: Mitral valve
NYHA: New York heart association classification
