# Supplementary material for: Prognostic value of hepatorenal function following transcatheter edge-to-edge mitral valve repair
Source: Clin Res Cardiol. 2021 Jul 12;110(12):1947–56. doi: 10.1007/s00392-021-01908-w (PMC8639570; doi:10.1007/s00392-021-01908-w)
Supplement: Supplementary file 1 — Supplementary file1 (DOCX 247 KB) [file 392_2021_1908_MOESM1_ESM.docx]

**Supplemental materials**

**Supplemental Figure 1. The composite outcome according to the MELD-XI score after TMVR in functional or degenerative MR.**


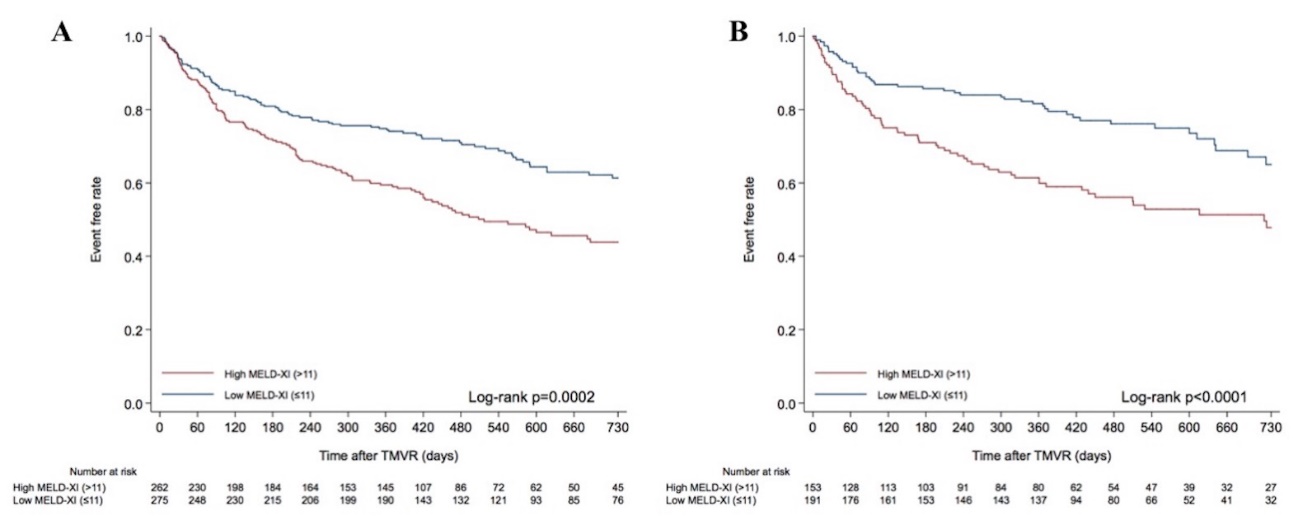


**Legends:** Kaplan-Meier curves demonstrating the composite outcome within 2 years after TMVR according to changes in NT-proBNP in functional MR (A) and degenerative MR (B).

**Supplemental Figure 2. Receiver-operating characteristic curve analysis for two-year composite outcome**

**
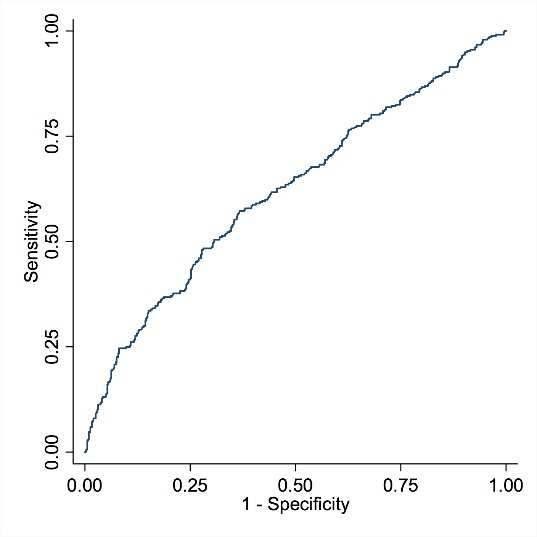
**

**Legends:** The receiver-operating characteristic curve analysis of the MELD-XI score for composite outcome within 2 year after TMVR (area under the curve: 0.62; sensitivity: 57%; specificity: 65%; positive predictive value: 49.1%; negative predictive value: 71%; p<0.0001).

**Supplemental Table 1. Periprocedural findings and clinical outcomes after TMVR**

|  | All | MELD-XI >11 | MELD-XI ≤11 |  |
| --- | --- | --- | --- | --- |
|  | n=881 | n=415 | n=466 | p value |
| Periprocedural findings |  |  |  |  |
| General anesthesia, n (%) | 657 (74.7) | 319 (77.1) | 337 (72.3) | 0.19 |
| Mean number of clips | 1.5 ± 0.6 | 1.6 ± 0.7 | 1.4 ± 0.6 | 0.001 |
| Implantation success, n (%) | 869 (98.6) | 409 (98.6) | 460 (98.7) | 1.00 |
| Post-procedural mean MVG (mmHg) | 3.9 ± 1.8 | 3.7 ± 1.7 | 4.1 ± 1.9 | 0.02 |
| Residual MR ≥3+, n (%) | 66 (7.5) | 32 (7.7) | 34 (7.3) | 0.81 |
| Length of stay (days) | 7 [6, 11] | 8 [6, 11] | 7 [5, 10] | 0.08 |
| Major or life-threating bleeding, n (%) | 52 (5.9) | 26 (6.3) | 26 (5.8) | 0.77 |
| Clinical outcomes |  |  |  |  |
| Composite outcome, n (%) | 337 (38.2) | 198 (47.7) | 139 (29.8) | <0.0001 |
| All-cause mortality, n (%) | 184 (20.9) | 116 (28.0) | 68 (14.6) | <0.0001 |
| HF hospitalization, n (%) | 203 (23.0) | 115 (27.7) | 88 (18.9) | 0.002 |
| Values shown are either n (%), mean ± SD, or median [interquartile range].  Legends: MELD-XI score = Model for End-stage Liver Disease excluding international normalized score; MVG = mitral valve gradient; MR = mitral regurgitation; HF = heart failure. | | | | |

**Supplemental Table 2. Changes in hepatorenal function after TMVR**

|  | Baseline | 1-month follow-up | 12-month follow-up | p value | | |
| --- | --- | --- | --- | --- | --- | --- |
|  | n=881 | n=254 | n=316 | baseline vs. 1 month | Baseline vs 12 months | 1 month vs 12 months |
| MELD-XI score | 11.0 ± 5.9 | 11.8 ± 6.3 | 11.2 ± 5.8 | 0.86 | 0.79 | 0.79 |
| High MELD-XI score (>11), n (%) | 415 (47.1) | 134 (52.8) | 151 (47.8) | 0.89* | 0.38* | 0.85* |
| eGFR (ml/min/1.73m^2^) | 48.0 [34.4, 63.1] | 44.4 [31.3, 59.9] | 47.2 [33.2, 61.1] | 0.12 | 0.15 | 0.74 |
| Total bilirubin (mg/dL) | 0.63 [0.44, 0.97] | 0.66 [0.43, 0.97] | 0.66 [ 0.41, 1.00] | 0.23 | 0.82 | 0.19 |
| Values shown are either n (%), mean ± SD or median [interquartile range]. * McNemar test was used for the comparison.  MELD-XI score = Model for End-stage Liver Disease excluding international normalized score; eGFR = estimated glomerular filtration rate. | | | | | | |

**Supplemental Table 3. The Harrell’s C-statistics for two-year composite outcome after TMVR**

|  | C-statistics | 95%CI | p value | p value  vs. MELD-XI score |
| --- | --- | --- | --- | --- |
| MELD-XI score | 0.60 | 0.57-0.63 | <0.0001 |  |
| eGFR (ml/min/1.73m^2^) | 0.57 | 0.54-0.61 | <0.0001 | 0.049 |
| Total Bilirubin (mg/dl) | 0.55 | 0.52-0.59 | <0.0001 | 0.004 |
| Legends: MELD-XI score = Model for End-stage Liver Disease excluding international normalized score, eGFR = estimated glomerular filtration rate. | | | | |

**Supplemental Table 4. Univariate analysis for predictors of the two-year composite outcome after TMVR**

|  | HR | 95%CI | p value |
| --- | --- | --- | --- |
| MELD-XI score >11 | 1.84 | 1.48 - 2.28 | <0.0001 |
| MELD- XI score per 1 increase | 1.06 | 1.04 - 1.08 | <0.0001 |
| Age (years) | 0.99 | 0.98 - 1.00 | 0.09 |
| Male | 1.25 | 1.01 – 1.56 | 0.047 |
| Diabetes mellitus | 1.33 | 1.05 - 1.66 | 0.02 |
| COPD | 1.48 | 1.15 - 1.89 | 0.003 |
| CAD | 1.28 | 1.02 - 1.60 | 0.03 |
| Previous CABG | 1.22 | 0.96 - 1.53 | 0.103 |
| Atrial fibrillation | 0.96 | 0.77 - 1.20 | 0.73 |
| NYHA class Ⅲ/Ⅳ | 1.86 | 1.34 - 2.66 | 0.0001 |
| Anemia | 1.61 | 1.29 - 2.02 | <0.0001 |
| Logistic Euro Score | 1.01 | 1.01 - 1.02 | <0.0001 |
| ICD | 1.33 | 0.99 - 1.74 | 0.06 |
| CRT | 1.21 | 0.85 - 1.67 | 0.29 |
| Hemodialysis | 1.36 | 0.65 - 2.49 | 0.39 |
| Functional MR | 1.20 | 0.96 - 1.50 | 0.12 |
| MR severity: 4+ | 1.24 | 0.90 - 1.67 | 0.18 |
| EROA (mm^2^) | 1.00 | 0.99 - 1.01 | 0.80 |
| LVEF ≤30% | 1.73 | 1.36 - 2.18 | <0.0001 |
| LVEDV per 10ml increase | 1.02 | 1.00 - 1.03 | 0.04 |
| RA area (mm^2^) | 1.00 | 0.99 - 1.01 | 0.44 |
| LA volume (ml) | 1.00 | 0.99 - 1.00 | 0.11 |
| TR severity: severe or more | 1.48 | 1.16 - 1.87 | 0.002 |
| TAPSE ≤14mm | 1.13 | 0.85 - 1.47 | 0.39 |
| SPAP (mmHg) | 1.00 | 0.99 - 1.01 | 0.75 |
| RAS inhibitor | 0.88 | 0.69 - 1.14 | 0.34 |
| Standardized furosemide equivalent per 10mg/day increase | 1.03 | 1.01 - 1.04 | 0.0002 |
| Device success | 0.76 | 0.51 - 1.17 | 0.20 |
| Legends: MELD-XI score = Model for End-stage Liver Disease excluding international normalized score, COPD = chronic obstructive pulmonary disease; CAD = coronary artery disease; CABG = coronary artery bypass graft; NYHA = New York Heart Association; EuroSCORE = European System for Cardiac Operative Risk Evaluation; ICD = implantable cardioverter defibrillator; CRT = cardiac resynchronization therapy; MR = mitral regurgitation; EROA = effective regurgitant orifice area; LVEF = left ventricular ejection fraction; LVEDV = left ventricular end-diastolic volume; RA = right atrium; LA = left atrium; TR = tricuspid regurgitation; TAPSE = tricuspid annular plane systolic excursion; SPAP = systolic pulmonary artery pressure; RAS = renin angiotensin system. | | | |
